# Editorial: Towards Long-Term Musculoskeletal Health Benefits in Adolescent Athletes: Specific Challenges in Primary and Secondary Prevention in This Pivotal Period

**DOI:** 10.3389/fspor.2022.830769

**Published:** 2022-02-07

**Authors:** Pascal Edouard, Kevin R. Ford

**Affiliations:** ^1^Inter-University Laboratory of Human Movement Sciences (LIBM EA 7424), University of Lyon, University Jean Monnet, Saint-Étienne, France; ^2^Sports Medicine Unit, Department of Clinical and Exercise Physiology, Faculty of Medicine, University Hospital of Saint-Etienne, Saint-Étienne, France; ^3^Department of Physical Therapy, Congdon School of Health Sciences, High Point University, High Point, NC, United States

**Keywords:** health protection, sports injury prevention, injury risk, sports rehabilitation, adolescent, youth–young adults

## Introduction

Reducing the risk of sports injury is of vital importance to support the numerous short- and long-term benefits athletes receive from sport participation (Edouard and Ford). This is especially true for adolescent athletes facing both rapid anatomical and physiological changes and increase in sport specialization as adolescents increase their training load, the number of competitions and expectations of them rise (Steffen and Engebretsen, 2010; Merkel, 2013; Bergeron et al., 2015; McKay et al., 2016). Additionally, risk factors for injury may emerge (McKay et al., 2016) which could be targeted with structured interventions aimed at reducing devastating injuries (Emery et al., 2015). Therefore, an adolescent-specific approach in primary and secondary prevention is warranted. In this context, this Research Topic aims to collect papers that address the unique challenges that emerge during adolescence which likely influence sports injuries. The six articles published in this Research Topic cover three of the four steps of the sequence of prevention from van Mechelen et al. (1992), and provide also relevant information about the injury context as suggested by Bolling et al. (2018).

## Epidemiology of Sports Injuries in Adolescent Athletes

In young female Swedish competitive figure skaters, Jederström et al. performed a cross-sectional study using an online questionnaire and reported that one-third of young female figure skaters had sustained a severe sports injury episode during the past year, and a fifth reported an ongoing injury episode. Overuse was the most common cause, and the lower limbs (knee, ankle and hip-groin) were the most common injury locations.

In adolescent distance runners, Mann et al. also performed a cross-sectional study using an online questionnaire to better understand epidemiology of running-related injury and training practices. Of the 113 athletes that responded, two-third sustained at least one new running-related injury during the 12-month study period, including self-reported pains or discomforts, representing an overall incidence rate was 6.3/1,000 participation hours (95% CI: 5.3–7.4). The knee, the foot/toes and the lower leg were the most involved injury locations, and overuse was the most common cause of injuries. The number of training sessions per week increased with chronological age and about two-third of athletes self-reported a high level of specialization.

These two studies provide relevant information about the injury risk in young female Swedish competitive figure skaters (Jederström et al.) and adolescent distance runners (Mann et al.) which will be of great help to orient injury risk reduction measures in research and development.

## Risk Factors of Sports Injuries in Adolescent Athletes

Understanding the sports injury risk factors represent one of the key for the development of appropriate injury risk reduction strategies (van Mechelen et al., 1992).

Jederström et al. reported that older age and an increased number of skipped meals per week were associated with increased injury risk. They concluded that long-term monotonous physical loads with increasing intensity and insufficient energy intake appear to predispose for injury in young female figure skaters. They also proposed a theoretical representation of health issues in female Swedish figure skaters for severe sports injuries with a multifactorial physical, psychological and societal approach.

Given the important number of injuries in elite youth soccer players (Pfirrmann et al., 2016). Kolodziej et al. analyzed in a prospective cohort study the association of neuromuscular laboratory-based performance parameters with the risk of non-contact lower extremity injuries in elite youth soccer players, using a decision tree model for a multivariate approach. 62 elite youth soccer players performed several neuromuscular tests at the start of the season and were followed for non-contact lower during a 10-months period of the season. Although this finding has to be taken with caution due to a small number of players and injury cases, (Kolodziej et al.) reported that measuring static postural control, postural control under unstable condition and thigh strength seem to enable a good indication of injury risk in elite youth soccer players.

Such findings (Jederström et al.; Kolodziej et al.) are of interest for the development of adolescent-specific injury risk reduction measures.

## Prevention Measures of Sports Injuries in Adolescent Athletes

Neuromuscular training programs are suggested to reduce the risk of sports injuries (Emery et al., 2015) and can also provide benefits for sports performance. However, adherence to such programs in real life is poor. Therefore, Räisänen et al. and Shill et al. aimed to describe coaches' injury prevention-related knowledge, attitudes, beliefs, and information sources, in youth basketball and Canadian high school rugby, respectively. For both, most of the coaches agreed with the importance of the need of sports injury risk reduction, and parts of neuromuscular training programs were used as warm-up, but balance exercises were not well adopted. They concluded that education on sports injury risk reduction should be improved, especially for coaches who are identified as the most common source of information for players (Räisänen et al.; Shill et al.).

Given the impact of running-related injuries in adolescent (Mann et al.). McSweeney et al. provides, through a narrative review and expert opinion, the state of knowledge and future directions for research in adolescent running biomechanics, injury prevention and supplemental training. They reported that limited evidence exists on the influence of biomechanics on running-related injury risk during growth and development in adolescent runners, expect prior injury and sex (female athletes); other potential risk factors associated with running-related injuries deserve further research. Evidence is also limited regarding recommendations for training in such age category. They concluded that comprehensive longitudinal studies monitoring changes in running biomechanics and associated musculoskeletal tissue are missing in adolescent runners (McSweeney et al.).

## Conclusions

This Research Topic provides new insights to better understand sports injuries in youth and adolescent athletes with relevant perspectives to reduce their risk. Efforts should continue by embracing the multifactorial nature of the sports injuries (Bittencourt et al., 2016) and the complexity of research on sports injury risk reduction (Edouard and Ford).

## Author Contributions

All authors listed have made a substantial, direct, and intellectual contribution to the work and approved it for publication.

## Conflict of Interest

The authors declare that the research was conducted in the absence of any commercial or financial relationships that could be construed as a potential conflict of interest.

## Publisher's Note

All claims expressed in this article are solely those of the authors and do not necessarily represent those of their affiliated organizations, or those of the publisher, the editors and the reviewers. Any product that may be evaluated in this article, or claim that may be made by its manufacturer, is not guaranteed or endorsed by the publisher.
